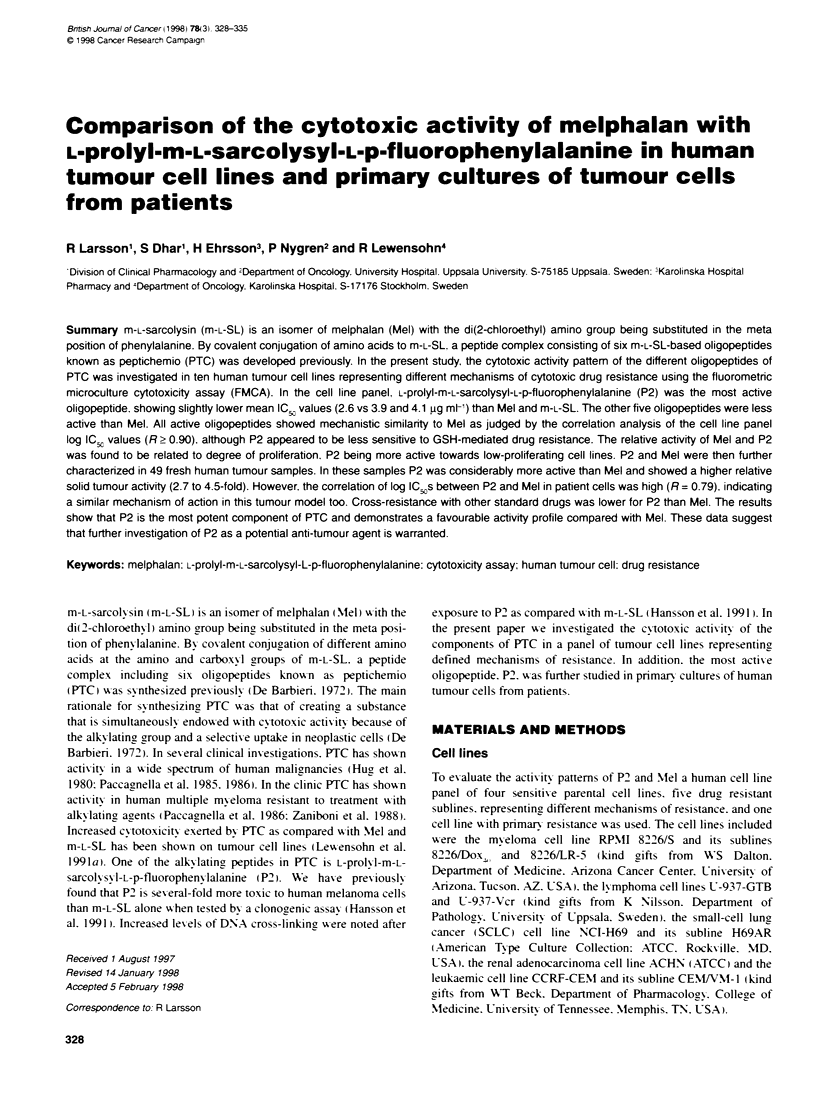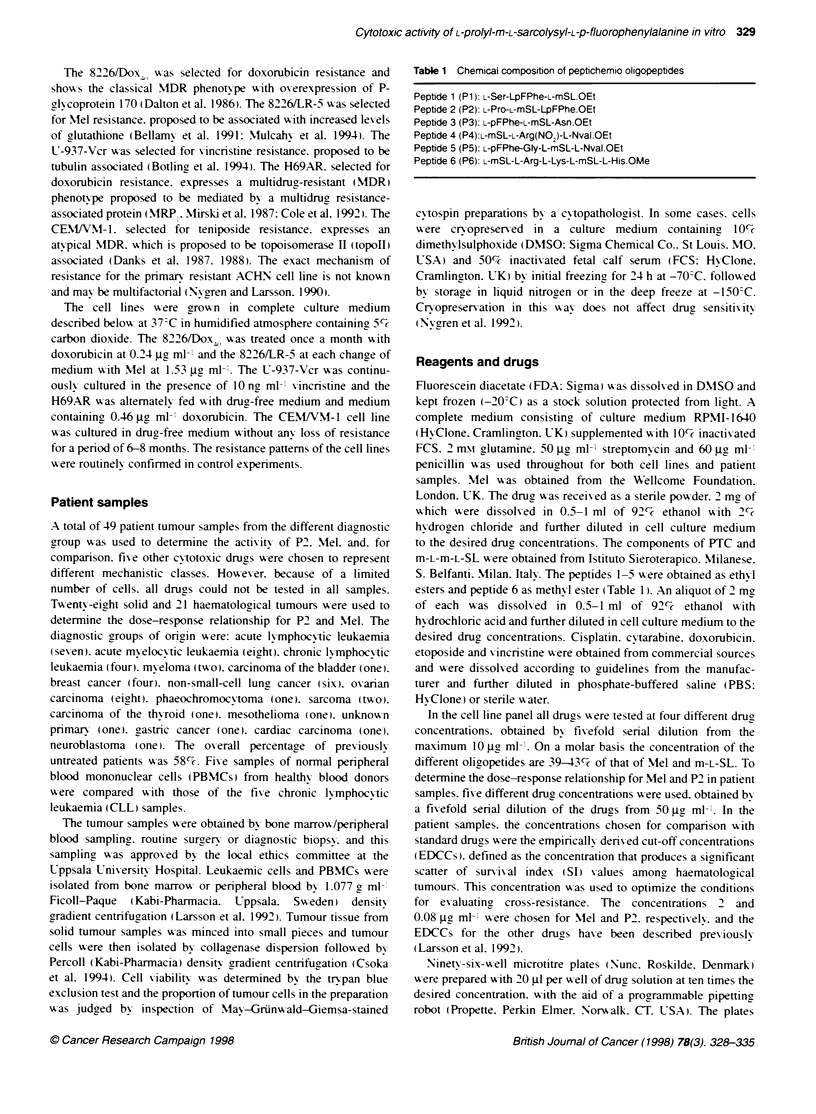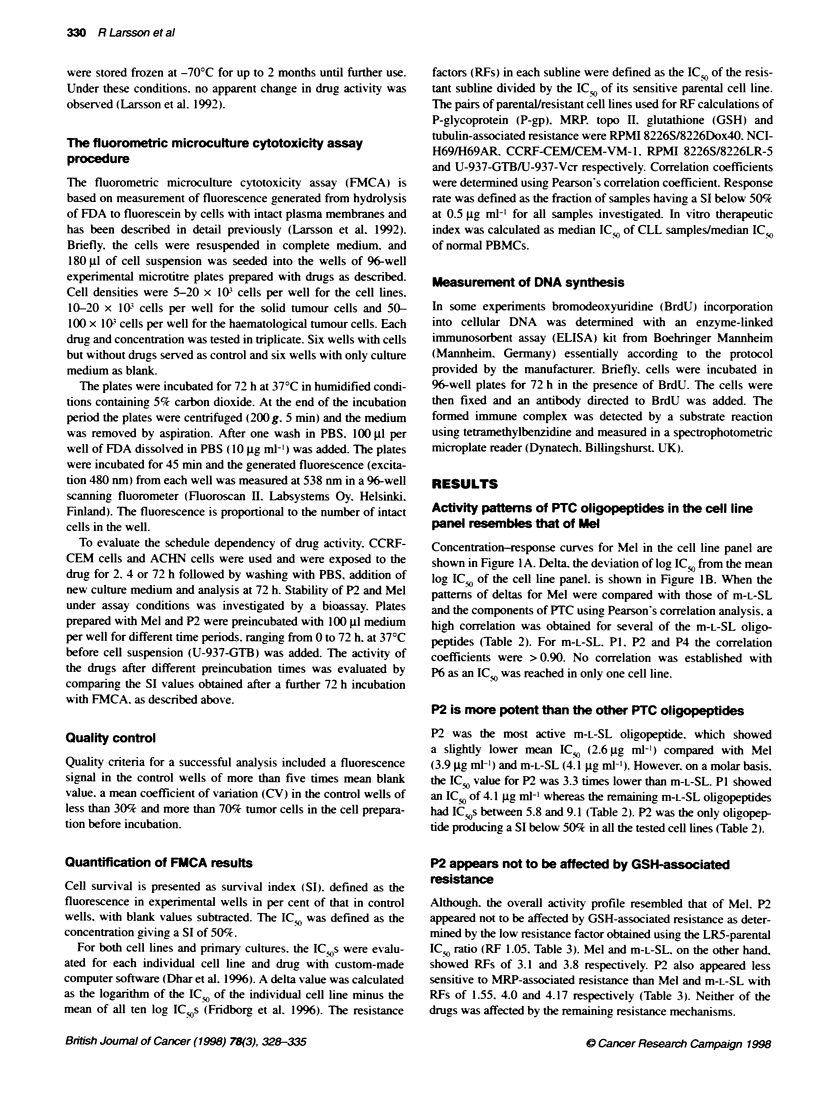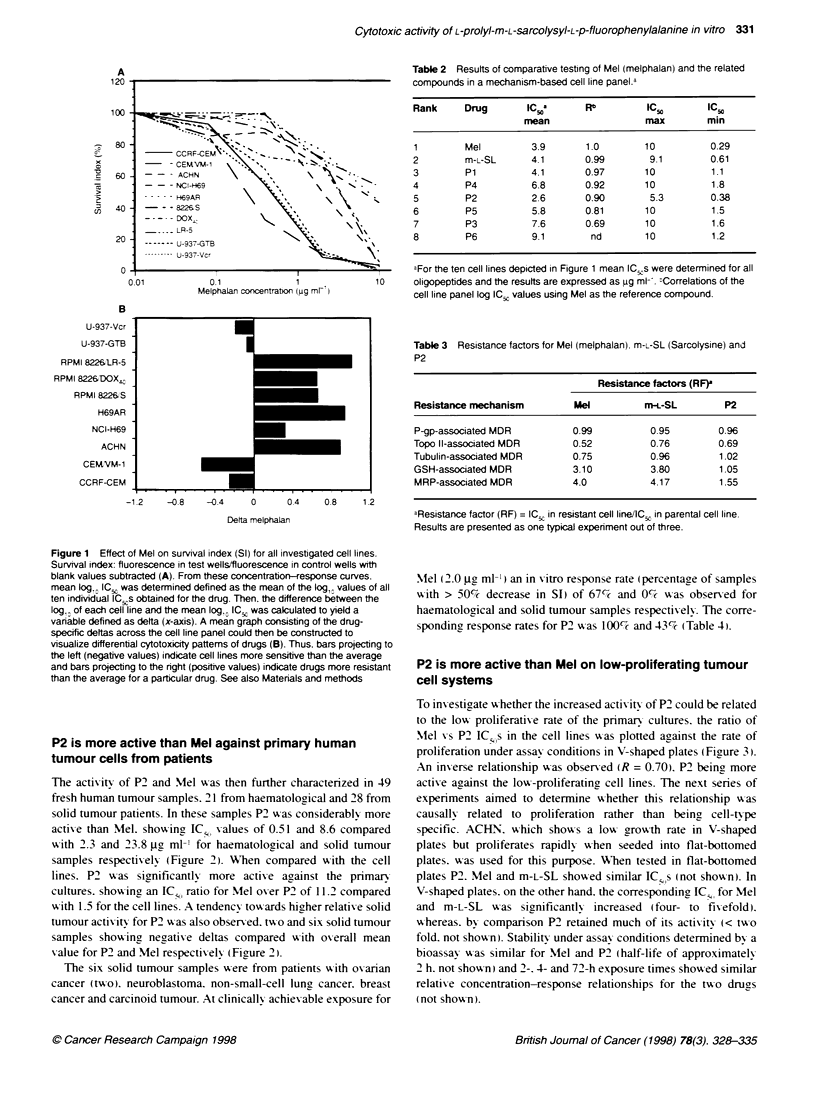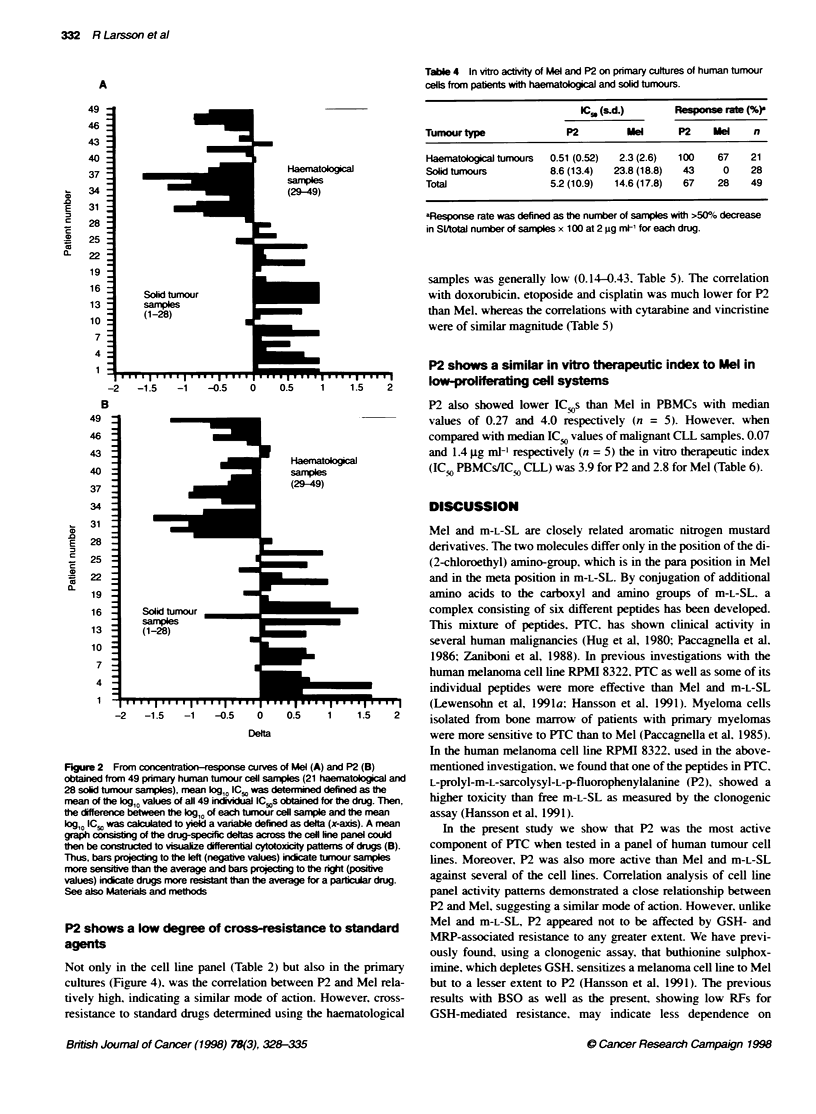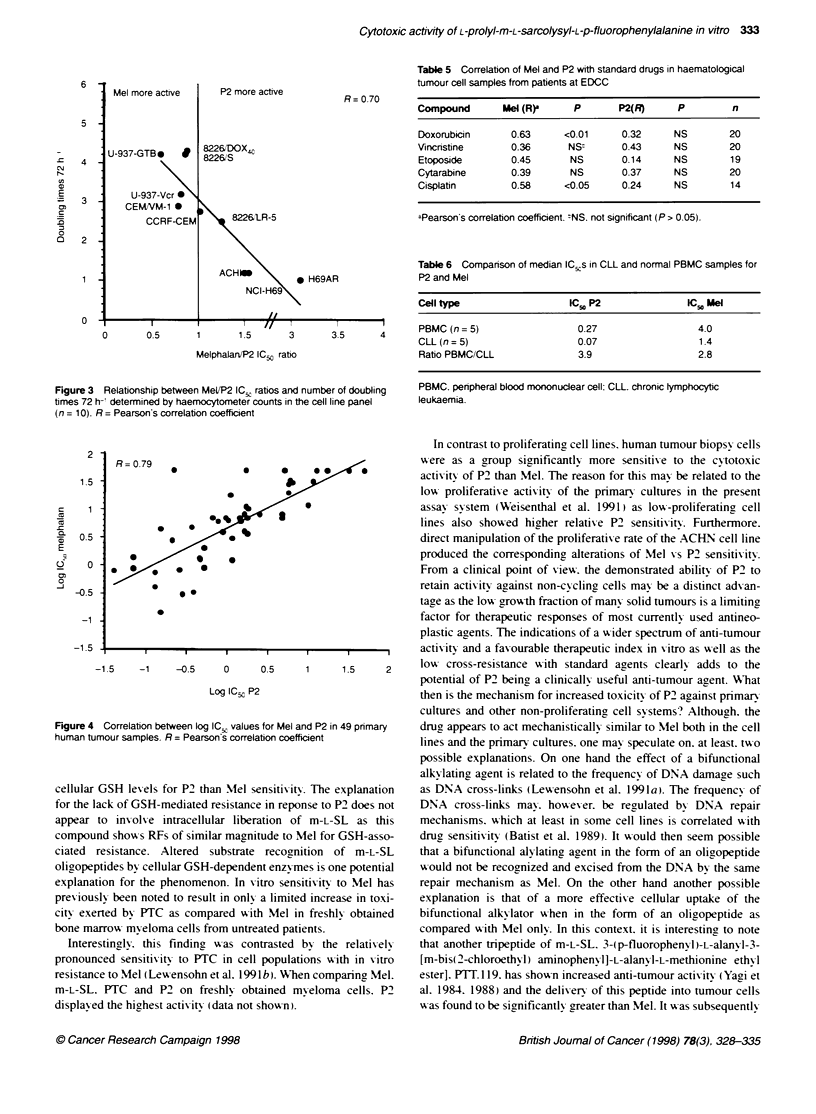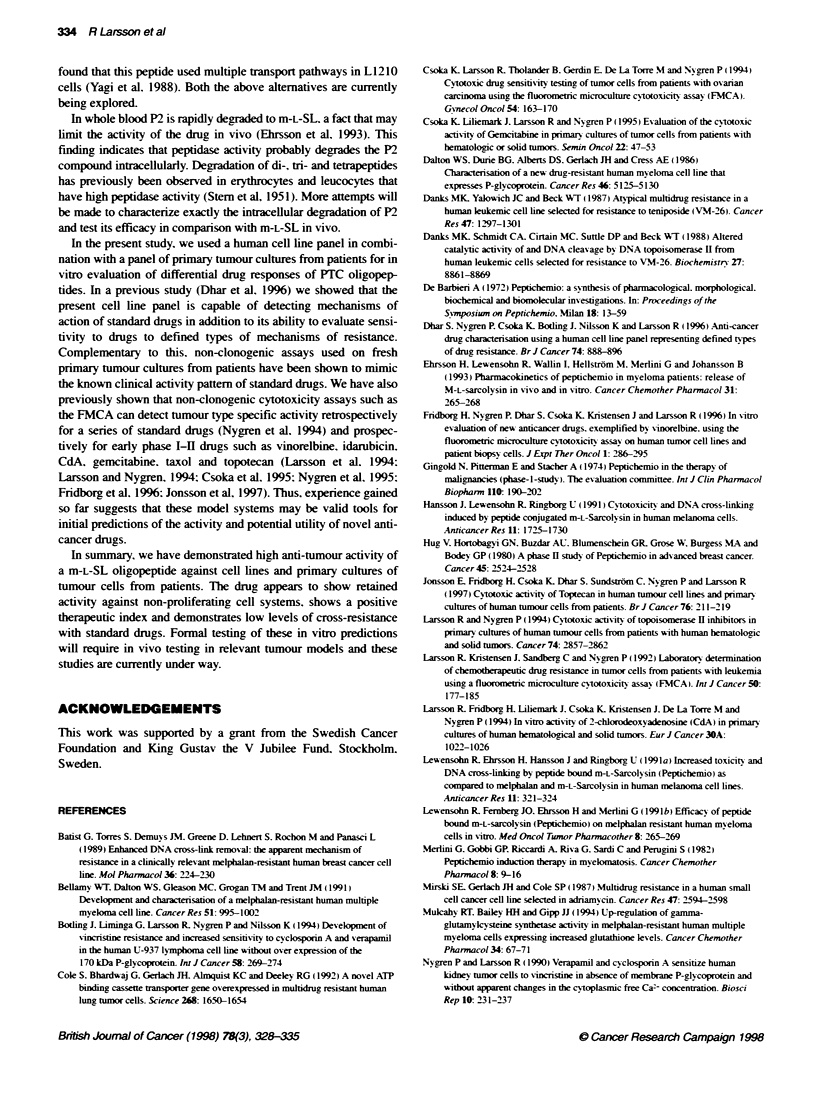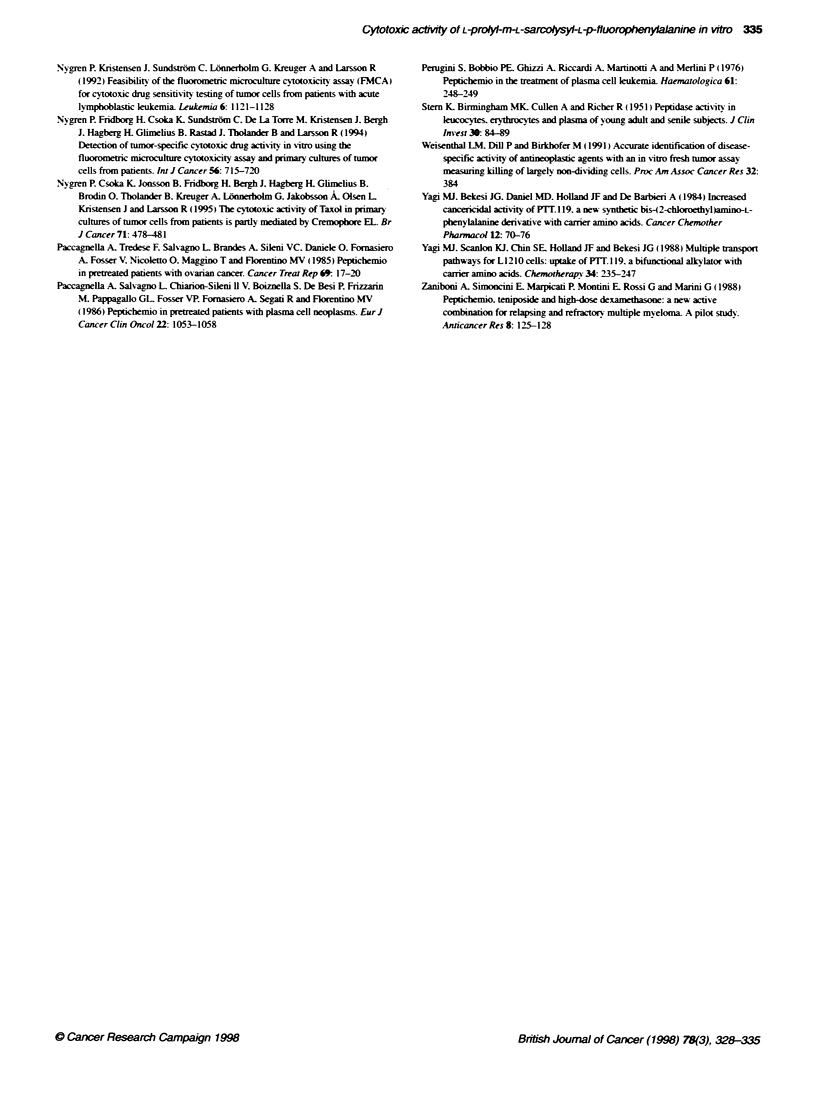# Comparison of the cytotoxic activity of melphalan with L-prolyl-m-L-sarcolysyl-L-p-fluorophenylalanine in human tumour cell lines and primary cultures of tumour cells from patients.

**DOI:** 10.1038/bjc.1998.494

**Published:** 1998-08

**Authors:** R. Larsson, S. Dhar, H. Ehrsson, P. Nygren, R. Lewensohn

**Affiliations:** Division of Clinical Pharmacology, University Hospital, Uppsala University, Sweden.

## Abstract

m-L-sarcolysin (m-L-SL) is an isomer of melphalan (Mel) with the di(2-chloroethyl) amino group being substituted in the meta position of phenylalanine. By covalent conjugation of amino acids to m-L-SL, a peptide complex consisting of six m-L-SL-based oligopeptides known as peptichemio (PTC) was developed previously. In the present study, the cytotoxic activity pattern of the different oligopeptides of PTC was investigated in ten human tumour cell lines representing different mechanisms of cytotoxic drug resistance using the fluorometric microculture cytotoxicity assay (FMCA). In the cell line panel, L-prolyl-m-L-sarcolysyl-L-p-fluorophenylalanine (P2) was the most active oligopeptide, showing slightly lower mean IC50 values (2.6 vs 3.9 and 4.1 microg ml(-1)) than Mel and m-L-SL. The other five oligopeptides were less active than Mel. All active oligopeptides showed mechanistic similarity to Mel as judged by the correlation analysis of the cell line panel log IC50 values (R > or = 0.90), although P2 appeared to be less sensitive to GSH-mediated drug resistance. The relative activity of Mel and P2 was found to be related to degree of proliferation, P2 being more active towards low-proliferating cell lines. P2 and Mel were then further characterized in 49 fresh human tumour samples. In these samples P2 was considerably more active than Mel and showed a higher relative solid tumour activity (2.7 to 4.5-fold). However, the correlation of log IC50s between P2 and Mel in patient cells was high (R = 0.79), indicating a similar mechanism of action in this tumour model too. Cross-resistance with other standard drugs was lower for P2 than Mel. The results show that P2 is the most potent component of PTC and demonstrates a favourable activity profile compared with Mel. These data suggest that further investigation of P2 as a potential anti-tumour agent is warranted.


British Journal of Cancer (1998) 78(3), 328–335
© 1998 Cancer Research Campaign

### Comparison of the cytotoxic activity of melphalan with L-prolyl-m-L-sarcolysyl-L-p-fluorophenylalanine in human tumour cell lines and primary cultures of tumour cells from patients

**R Larsson[1], S Dhar[1], H Ehrsson[3], P Nygren[2] and R Lewensohn[4]**

[1]Division of Clinical Pharmacology and [2]Department of Oncology, University Hospital, Uppsala University, S-75185 Uppsala, Sweden; [3]Karolinska Hospital Pharmacy and [4]Department of Oncology, Karolinska Hospital, S-17176 Stockholm, Sweden

**Summary** m-L-sarcolysin (m-L-SL) is an isomer of melphalan (Mel) with the di(2-chloroethyl) amino group being substituted in the meta position of phenylalanine. By covalent conjugation of amino acids to m-L-SL, a peptide complex consisting of six m-L-SL-based oligopeptides known as peptichemio (PTC) was developed previously. In the present study, the cytotoxic activity pattern of the different oligopeptides of PTC was investigated in ten human tumour cell lines representing different mechanisms of cytotoxic drug resistance using the fluorometric microculture cytotoxicity assay (FMCA). In the cell line panel, L-prolyl-m-L-sarcolysyl-L-p-fluorophenylalanine (P2) was the most active oligopeptide, showing slightly lower mean $IC_{50}$ values (2.6 vs 3.9 and 4.1 μg $ml^{-1}$) than Mel and m-L-SL. The other five oligopeptides were less active than Mel. All active oligopeptides showed mechanistic similarity to Mel as judged by the correlation analysis of the cell line panel log $IC_{50}$ values ($R \geq 0.90$), although P2 appeared to be less sensitive to GSH-mediated drug resistance. The relative activity of Mel and P2 was found to be related to degree of proliferation, P2 being more active towards low-proliferating cell lines. P2 and Mel were then further characterized in 49 fresh human tumour samples. In these samples P2 was considerably more active than Mel and showed a higher relative solid tumour activity (2.7 to 4.5-fold). However, the correlation of log $IC_{50}$s between P2 and Mel in patient cells was high ($R = 0.79$), indicating a similar mechanism of action in this tumour model too. Cross-resistance with other standard drugs was lower for P2 than Mel. The results show that P2 is the most potent component of PTC and demonstrates a favourable activity profile compared with Mel. These data suggest that further investigation of P2 as a potential anti-tumour agent is warranted.

**Keywords:** melphalan; L-prolyl-m-L-sarcolysyl-L-p-fluorophenylalanine; cytotoxicity assay; human tumour cell; drug resistance m-L-sarcolysin (m-L-SL) is an isomer of melphalan (Mel) with the di(2-chloroethyl) amino group being substituted in the meta position of phenylalanine. By covalent conjugation of different amino acids at the amino and carboxyl groups of m-L-SL, a peptide complex including six oligopeptides known as peptichemio (PTC) was synthesized previously (De Barbieri, 1972). The main rationale for synthesizing PTC was that of creating a substance that is simultaneously endowed with cytotoxic activity because of the alkylating group and a selective uptake in neoplastic cells (De Barbieri, 1972). In several clinical investigations, PTC has shown activity in a wide spectrum of human malignancies (Hug et al, 1980; Paccagnella et al, 1985, 1986). In the clinic PTC has shown activity in human multiple myeloma resistant to treatment with alkylating agents (Paccagnella et al, 1986; Zaniboni et al, 1988). Increased cytotoxicity exerted by PTC as compared with Mel and m-L-SL has been shown on tumour cell lines (Lewensohn et al, 1991*a*). One of the alkylating peptides in PTC is L-prolyl-m-L-sarcolysyl-L-p-fluorophenylalanine (P2). We have previously found that P2 is several-fold more toxic to human melanoma cells than m-L-SL alone when tested by a clonogenic assay (Hansson et al, 1991). Increased levels of DNA cross-linking were noted after exposure to P2 as compared with m-L-SL (Hansson et al, 1991). In the present paper we investigated the cytotoxic activity of the components of PTC in a panel of tumour cell lines representing defined mechanisms of resistance. In addition, the most active oligopeptide, P2, was further studied in primary cultures of human tumour cells from patients.

*Received 1 August 1997*
*Revised 14 January 1998*
*Accepted 5 February 1998*

*Correspondence to:* R Larsson

### MATERIALS AND METHODS

### Cell lines

To evaluate the activity patterns of P2 and Mel a human cell line panel of four sensitive parental cell lines, five drug resistant sublines, representing different mechanisms of resistance, and one cell line with primary resistance was used. The cell lines included were the myeloma cell line RPMI 8226/S and its sublines 8226/$Dox_{40}$ and 8226/LR-5 (kind gifts from WS Dalton, Department of Medicine, Arizona Cancer Center, University of Arizona, Tucson, AZ, USA), the lymphoma cell lines U-937-GTB and U-937-Vcr (kind gifts from K Nilsson, Department of Pathology, University of Uppsala, Sweden), the small-cell lung cancer (SCLC) cell line NCI-H69 and its subline H69AR (American Type Culture Collection; ATCC, Rockville, MD, USA), the renal adenocarcinoma cell line ACHN (ATCC) and the leukaemic cell line CCRF-CEM and its subline CEM/VM-1 (kind gifts from WT Beck, Department of Pharmacology, College of Medicine, University of Tennessee, Memphis, TN, USA).

The 8226/$Dox_{40}$ was selected for doxorubicin resistance and shows the classical MDR phenotype with overexpression of P-glycoprotein 170 (Dalton et al. 1986). The 8226/LR-5 was selected for Mel resistance, proposed to be associated with increased levels of glutathione (Bellamy et al. 1991; Mulcahy et al. 1994). The U-937-Vcr was selected for vincristine resistance, proposed to be tubulin associated (Botling et al. 1994). The H69AR, selected for doxorubicin resistance, expresses a multidrug-resistant (MDR) phenotype proposed to be mediated by a multidrug resistance-associated protein ($MRP_1$, Mirski et al. 1987; Cole et al. 1992). The CEM/VM-1, selected for teniposide resistance, expresses an atypical MDR, which is proposed to be topoisomerase II (topoII) associated (Danks et al. 1987, 1988). The exact mechanism of resistance for the primary resistant ACHN cell line is not known and may be multifactorial (Nygren and Larsson, 1990).

The cell lines were grown in complete culture medium described below at 37°C in humidified atmosphere containing 5% carbon dioxide. The 8226/$Dox_{40}$ was treated once a month with doxorubicin at 0.24 µg $ml^{-1}$ and the 8226/LR-5 at each change of medium with Mel at 1.53 µg $ml^{-1}$. The U-937-Vcr was continuously cultured in the presence of 10 ng $ml^{-1}$ vincristine and the H69AR was alternately fed with drug-free medium and medium containing 0.46 µg $ml^{-1}$ doxorubicin. The CEM/VM-1 cell line was cultured in drug-free medium without any loss of resistance for a period of 6–8 months. The resistance patterns of the cell lines were routinely confirmed in control experiments.

### Patient samples

A total of 49 patient tumour samples from the different diagnostic group was used to determine the activity of P2, Mel, and, for comparison, five other cytotoxic drugs were chosen to represent different mechanistic classes. However, because of a limited number of cells, all drugs could not be tested in all samples. Twenty-eight solid and 21 haematological tumours were used to determine the dose–response relationship for P2 and Mel. The diagnostic groups of origin were: acute lymphocytic leukaemia (seven), acute myelocytic leukaemia (eight), chronic lymphocytic leukaemia (four), myeloma (two), carcinoma of the bladder (one), breast cancer (four), non-small-cell lung cancer (six), ovarian carcinoma (eight), phaeochromocytoma (one), sarcoma (two), carcinoma of the thyroid (one), mesothelioma (one), unknown primary (one), gastric cancer (one), cardiac carcinoma (one), neuroblastoma (one). The overall percentage of previously untreated patients was 58%. Five samples of normal peripheral blood mononuclear cells (PBMCs) from healthy blood donors were compared with those of the five chronic lymphocytic leukaemia (CLL) samples.

The tumour samples were obtained by bone marrow/peripheral blood sampling, routine surgery or diagnostic biopsy, and this sampling was approved by the local ethics committee at the Uppsala University Hospital. Leukaemic cells and PBMCs were isolated from bone marrow or peripheral blood by 1.077 g $ml^{-1}$ Ficoll–Paque (Kabi-Pharmacia, Uppsala, Sweden) density gradient centrifugation (Larsson et al. 1992). Tumour tissue from solid tumour samples was minced into small pieces and tumour cells were then isolated by collagenase dispersion followed by Percoll (Kabi-Pharmacia) density gradient centrifugation (Csoka et al. 1994). Cell viability was determined by the trypan blue exclusion test and the proportion of tumour cells in the preparation was judged by inspection of May–Grünwald–Giemsa-stained cytospin preparations by a cytopathologist. In some cases, cells were cryopreserved in a culture medium containing 10% dimethylsulphoxide (DMSO; Sigma Chemical Co., St Louis, MO, USA) and 50% inactivated fetal calf serum (FCS; HyClone, Cramlington, UK) by initial freezing for 24 h at −70°C, followed by storage in liquid nitrogen or in the deep freeze at −150°C. Cryopreservation in this way does not affect drug sensitivity (Nygren et al. 1992).

**Table 1** Chemical composition of peptichemio oligopeptides

| |
|---|
| Peptide 1 (P1): L-Ser-LpFPhe-L-mSL.OEt |
| Peptide 2 (P2): L-Pro-L-mSL-LpFPhe.OEt |
| Peptide 3 (P3): L-pFPhe-L-mSL-Asn.OEt |
| Peptide 4 (P4):L-mSL-L-Arg($NO_2$)-L-Nval.OEt |
| Peptide 5 (P5): L-pFPhe-Gly-L-mSL-L-Nval.OEt |
| Peptide 6 (P6): L-mSL-L-Arg-L-Lys-L-mSL-L-His.OMe |

### Reagents and drugs

Fluorescein diacetate (FDA; Sigma) was dissolved in DMSO and kept frozen (−20°C) as a stock solution protected from light. A complete medium consisting of culture medium RPMI-1640 (HyClone, Cramlington, UK) supplemented with 10% inactivated FCS, 2 mM glutamine, 50 µg $ml^{-1}$ streptomycin and 60 µg $ml^{-1}$ penicillin was used throughout for both cell lines and patient samples. Mel was obtained from the Wellcome Foundation, London, UK. The drug was received as a sterile powder, 2 mg of which were dissolved in 0.5–1 ml of 92% ethanol with 2% hydrogen chloride and further diluted in cell culture medium to the desired drug concentrations. The components of PTC and m-L-m-L-SL were obtained from Istituto Sieroterapico, Milanese, S. Belfanti, Milan, Italy. The peptides 1–5 were obtained as ethyl esters and peptide 6 as methyl ester (Table 1). An aliquot of 2 mg of each was dissolved in 0.5–1 ml of 92% ethanol with hydrochloric acid and further diluted in cell culture medium to the desired drug concentrations. Cisplatin, cytarabine, doxorubicin, etoposide and vincristine were obtained from commercial sources and were dissolved according to guidelines from the manufacturer and further diluted in phosphate-buffered saline (PBS; HyClone) or sterile water.

In the cell line panel all drugs were tested at four different drug concentrations, obtained by fivefold serial dilution from the maximum 10 µg $ml^{-1}$. On a molar basis the concentration of the different oligopetides are 39–43% of that of Mel and m-L-SL. To determine the dose–response relationship for Mel and P2 in patient samples, five different drug concentrations were used, obtained by a fivefold serial dilution of the drugs from 50 µg $ml^{-1}$. In the patient samples, the concentrations chosen for comparison with standard drugs were the empirically derived cut-off concentrations (EDCCs), defined as the concentration that produces a significant scatter of survival index (SI) values among haematological tumours. This concentration was used to optimize the conditions for evaluating cross-resistance. The concentrations 2 and 0.08 µg $ml^{-1}$ were chosen for Mel and P2, respectively, and the EDCCs for the other drugs have been described previously (Larsson et al. 1992).

Ninety-six-well microtitre plates (Nunc, Roskilde, Denmark) were prepared with 20 µl per well of drug solution at ten times the desired concentration, with the aid of a programmable pipetting robot (Propette, Perkin Elmer, Norwalk, CT, USA). The plates

© Cancer Research Campaign 1998

were stored frozen at –70°C for up to 2 months until further use. Under these conditions, no apparent change in drug activity was observed (Larsson et al, 1992).

### The fluorometric microculture cytotoxicity assay procedure

The fluorometric microculture cytotoxicity assay (FMCA) is based on measurement of fluorescence generated from hydrolysis of FDA to fluorescein by cells with intact plasma membranes and has been described in detail previously (Larsson et al, 1992). Briefly, the cells were resuspended in complete medium, and 180 μl of cell suspension was seeded into the wells of 96-well experimental microtitre plates prepared with drugs as described. Cell densities were 5–20 × $10^3$ cells per well for the cell lines, 10–20 × $10^3$ cells per well for the solid tumour cells and 50–100 × $10^3$ cells per well for the haematological tumour cells. Each drug and concentration was tested in triplicate. Six wells with cells but without drugs served as control and six wells with only culture medium as blank.

The plates were incubated for 72 h at 37°C in humidified conditions containing 5% carbon dioxide. At the end of the incubation period the plates were centrifuged (200 *g*, 5 min) and the medium was removed by aspiration. After one wash in PBS, 100 μl per well of FDA dissolved in PBS (10 μg $ml^{-1}$) was added. The plates were incubated for 45 min and the generated fluorescence (excitation 480 nm) from each well was measured at 538 nm in a 96-well scanning fluorometer (Fluoroscan II, Labsystems Oy, Helsinki, Finland). The fluorescence is proportional to the number of intact cells in the well.

To evaluate the schedule dependency of drug activity, CCRF-CEM cells and ACHN cells were used and were exposed to the drug for 2, 4 or 72 h followed by washing with PBS, addition of new culture medium and analysis at 72 h. Stability of P2 and Mel under assay conditions was investigated by a bioassay. Plates prepared with Mel and P2 were preincubated with 100 μl medium per well for different time periods, ranging from 0 to 72 h, at 37°C before cell suspension (U-937-GTB) was added. The activity of the drugs after different preincubation times was evaluated by comparing the SI values obtained after a further 72 h incubation with FMCA, as described above.

### Quality control

Quality criteria for a successful analysis included a fluorescence signal in the control wells of more than five times mean blank value, a mean coefficient of variation (CV) in the control wells of less than 30% and more than 70% tumor cells in the cell preparation before incubation.

### Quantification of FMCA results

Cell survival is presented as survival index (SI), defined as the fluorescence in experimental wells in per cent of that in control wells, with blank values subtracted. The $IC_{50}$ was defined as the concentration giving a SI of 50%.

For both cell lines and primary cultures, the $IC_{50}$s were evaluated for each individual cell line and drug with custom-made computer software (Dhar et al, 1996). A delta value was calculated as the logarithm of the $IC_{50}$ of the individual cell line minus the mean of all ten log $IC_{50}$s (Fridborg et al, 1996). The resistance factors (RFs) in each subline were defined as the $IC_{50}$ of the resistant subline divided by the $IC_{50}$ of its sensitive parental cell line. The pairs of parental/resistant cell lines used for RF calculations of P-glycoprotein (P-gp), MRP, topo II, glutathione (GSH) and tubulin-associated resistance were RPMI 8226S/8226Dox40, NCI-H69/H69AR, CCRF-CEM/CEM-VM-1, RPMI 8226S/8226LR-5 and U-937-GTB/U-937-Vcr respectively. Correlation coefficients were determined using Pearson's correlation coefficient. Response rate was defined as the fraction of samples having a SI below 50% at 0.5 μg $ml^{-1}$ for all samples investigated. In vitro therapeutic index was calculated as median $IC_{50}$ of CLL samples/median $IC_{50}$ of normal PBMCs.

### Measurement of DNA synthesis

In some experiments bromodeoxyuridine (BrdU) incorporation into cellular DNA was determined with an enzyme-linked immunosorbent assay (ELISA) kit from Boehringer Mannheim (Mannheim, Germany) essentially according to the protocol provided by the manufacturer. Briefly, cells were incubated in 96-well plates for 72 h in the presence of BrdU. The cells were then fixed and an antibody directed to BrdU was added. The formed immune complex was detected by a substrate reaction using tetramethylbenzidine and measured in a spectrophotometric microplate reader (Dynatech, Billingshurst, UK).

### RESULTS

### Activity patterns of PTC oligopeptides in the cell line panel resembles that of Mel

Concentration–response curves for Mel in the cell line panel are shown in Figure 1A. Delta, the deviation of log $IC_{50}$ from the mean log $IC_{50}$ of the cell line panel, is shown in Figure 1B. When the patterns of deltas for Mel were compared with those of m-L-SL and the components of PTC using Pearson's correlation analysis, a high correlation was obtained for several of the m-L-SL oligopeptides (Table 2). For m-L-SL, P1, P2 and P4 the correlation coefficients were > 0.90. No correlation was established with P6 as an $IC_{50}$ was reached in only one cell line.

### P2 is more potent than the other PTC oligopeptides

P2 was the most active m-L-SL oligopeptide, which showed a slightly lower mean $IC_{50}$ (2.6 μg $ml^{-1}$) compared with Mel (3.9 μg $ml^{-1}$) and m-L-SL (4.1 μg $ml^{-1}$). However, on a molar basis, the $IC_{50}$ value for P2 was 3.3 times lower than m-L-SL. P1 showed an $IC_{50}$ of 4.1 μg $ml^{-1}$ whereas the remaining m-L-SL oligopeptides had $IC_{50}$s between 5.8 and 9.1 (Table 2). P2 was the only oligopeptide producing a SI below 50% in all the tested cell lines (Table 2).

### P2 appears not to be affected by GSH-associated resistance

Although, the overall activity profile resembled that of Mel, P2 appeared not to be affected by GSH-associated resistance as determined by the low resistance factor obtained using the LR5-parental $IC_{50}$ ratio (RF 1.05, Table 3). Mel and m-L-SL, on the other hand, showed RFs of 3.1 and 3.8 respectively. P2 also appeared less sensitive to MRP-associated resistance than Mel and m-L-SL with RFs of 1.55, 4.0 and 4.17 respectively (Table 3). Neither of the drugs was affected by the remaining resistance mechanisms.

© Cancer Research Campaign 1998

**Figure 1** Effect of Mel on survival index (SI) for all investigated cell lines. Survival index: fluorescence in test wells/fluorescence in control wells with blank values subtracted (**A**). From these concentration–response curves, mean $\log_{10}$ $IC_{50}$ was determined defined as the mean of the $\log_{10}$ values of all ten individual $IC_{50}$s obtained for the drug. Then, the difference between the $\log_{10}$ of each cell line and the mean $\log_{10}$ $IC_{50}$ was calculated to yield a variable defined as delta (x-axis). A mean graph consisting of the drug-specific deltas across the cell line panel could then be constructed to visualize differential cytotoxicity patterns of drugs (**B**). Thus, bars projecting to the left (negative values) indicate cell lines more sensitive than the average and bars projecting to the right (positive values) indicate drugs more resistant than the average for a particular drug. See also Materials and methods

### P2 is more active than Mel against primary human tumour cells from patients

The activity of P2 and Mel was then further characterized in 49 fresh human tumour samples, 21 from haematological and 28 from solid tumour patients. In these samples P2 was considerably more active than Mel, showing $IC_{50}$ values of 0.51 and 8.6 compared with 2.3 and 23.8 µg $ml^{-1}$ for haematological and solid tumour samples respectively (Figure 2). When compared with the cell lines, P2 was significantly more active against the primary cultures, showing an $IC_{50}$ ratio for Mel over P2 of 11.2 compared with 1.5 for the cell lines. A tendency towards higher relative solid tumour activity for P2 was also observed, two and six solid tumour samples showing negative deltas compared with overall mean value for P2 and Mel respectively (Figure 2).

The six solid tumour samples were from patients with ovarian cancer (two), neuroblastoma, non-small-cell lung cancer, breast cancer and carcinoid tumour. At clinically achievable exposure for

**Table 2** Results of comparative testing of Mel (melphalan) and the related compounds in a mechanism-based cell line panel.[a]

| Rank | Drug | $IC_{50}$[a] mean | R[b] | $IC_{50}$ max | $IC_{50}$ min |
|---|---|---|---|---|---|
| 1 | Mel | 3.9 | 1.0 | 10 | 0.29 |
| 2 | m-L-SL | 4.1 | 0.99 | 9.1 | 0.61 |
| 3 | P1 | 4.1 | 0.97 | 10 | 1.1 |
| 4 | P4 | 6.8 | 0.92 | 10 | 1.8 |
| 5 | P2 | 2.6 | 0.90 | 5.3 | 0.38 |
| 6 | P5 | 5.8 | 0.81 | 10 | 1.5 |
| 7 | P3 | 7.6 | 0.69 | 10 | 1.6 |
| 8 | P6 | 9.1 | nd | 10 | 1.2 |

[a]For the ten cell lines depicted in Figure 1 mean $IC_{50}$s were determined for all oligopeptides and the results are expressed as µg $ml^{-1}$. [b]Correlations of the cell line panel log $IC_{50}$ values using Mel as the reference compound.

**Table 3** Resistance factors for Mel (melphalan), m-L-SL (Sarcolysine) and P2

| | Resistance factors (RF)[a] | | |
|---|---|---|---|
| Resistance mechanism | Mel | m-L-SL | P2 |
| P-gp-associated MDR | 0.99 | 0.95 | 0.96 |
| Topo II-associated MDR | 0.52 | 0.76 | 0.69 |
| Tubulin-associated MDR | 0.75 | 0.96 | 1.02 |
| GSH-associated MDR | 3.10 | 3.80 | 1.05 |
| MRP-associated MDR | 4.0 | 4.17 | 1.55 |

[a]Resistance factor (RF) = $IC_{50}$ in resistant cell line/$IC_{50}$ in parental cell line. Results are presented as one typical experiment out of three.

Mel (2.0 µg $ml^{-1}$) an in vitro response rate (percentage of samples with > 50% decrease in SI) of 67% and 0% was observed for haematological and solid tumour samples respectively. The corresponding response rates for P2 was 100% and 43% (Table 4).

### P2 is more active than Mel on low-proliferating tumour cell systems

To investigate whether the increased activity of P2 could be related to the low proliferative rate of the primary cultures, the ratio of Mel vs P2 $IC_{50}$s in the cell lines was plotted against the rate of proliferation under assay conditions in V-shaped plates (Figure 3). An inverse relationship was observed ($R$ = 0.70), P2 being more active against the low-proliferating cell lines. The next series of experiments aimed to determine whether this relationship was causally related to proliferation rather than being cell-type specific. ACHN, which shows a low growth rate in V-shaped plates but proliferates rapidly when seeded into flat-bottomed plates, was used for this purpose. When tested in flat-bottomed plates P2, Mel and m-L-SL showed similar $IC_{50}$s (not shown). In V-shaped plates, on the other hand, the corresponding $IC_{50}$ for Mel and m-L-SL was significantly increased (four- to fivefold), whereas, by comparison P2 retained much of its activity (< two fold, not shown). Stability under assay conditions determined by a bioassay was similar for Mel and P2 (half-life of approximately 2 h, not shown) and 2-, 4- and 72-h exposure times showed similar relative concentration–response relationships for the two drugs (not shown).

**Figure 2** From concentration–response curves of Mel (**A**) and P2 (**B**) obtained from 49 primary human tumour cell samples (21 haematological and 28 solid tumour samples), mean $\log_{10}$ $IC_{50}$ was determined defined as the mean of the $\log_{10}$ values of all 49 individual $IC_{50}$s obtained for the drug. Then, the difference between the $\log_{10}$ of each tumour cell sample and the mean $\log_{10}$ $IC_{50}$ was calculated to yield a variable defined as delta (*x*-axis). A mean graph consisting of the drug-specific deltas across the cell line panel could then be constructed to visualize differential cytotoxicity patterns of drugs (**B**). Thus, bars projecting to the left (negative values) indicate tumour samples more sensitive than the average and bars projecting to the right (positive values) indicate drugs more resistant than the average for a particular drug. See also Materials and methods

### P2 shows a low degree of cross-resistance to standard agents

Not only in the cell line panel (Table 2) but also in the primary cultures (Figure 4), was the correlation between P2 and Mel relatively high, indicating a similar mode of action. However, cross-resistance to standard drugs determined using the haematological samples was generally low (0.14–0.43, Table 5). The correlation with doxorubicin, etoposide and cisplatin was much lower for P2 than Mel, whereas the correlations with cytarabine and vincristine were of similar magnitude (Table 5)

**Table 4** In vitro activity of Mel and P2 on primary cultures of human tumour cells from patients with haematological and solid tumours.

| | $IC_{50}$ (s.d.) | | Response rate (%)[a] | | |
|---|---|---|---|---|---|
| **Tumour type** | **P2** | **Mel** | **P2** | **Mel** | ***n*** |
| Haematological tumours | 0.51 (0.52) | 2.3 (2.6) | 100 | 67 | 21 |
| Solid tumours | 8.6 (13.4) | 23.8 (18.8) | 43 | 0 | 28 |
| Total | 5.2 (10.9) | 14.6 (17.8) | 67 | 28 | 49 |

[a]Response rate was defined as the number of samples with >50% decrease in SI/total number of samples × 100 at 2 μg $ml^{-1}$ for each drug.

### P2 shows a similar in vitro therapeutic index to Mel in low-proliferating cell systems

P2 also showed lower $IC_{50}$s than Mel in PBMCs with median values of 0.27 and 4.0 respectively (*n* = 5). However, when compared with median $IC_{50}$ values of malignant CLL samples, 0.07 and 1.4 μg $ml^{-1}$ respectively (*n* = 5) the in vitro therapeutic index ($IC_{50}$ PBMCs/$IC_{50}$ CLL) was 3.9 for P2 and 2.8 for Mel (Table 6).

### DISCUSSION

Mel and m-L-SL are closely related aromatic nitrogen mustard derivatives. The two molecules differ only in the position of the di-(2-chloroethyl) amino-group, which is in the para position in Mel and in the meta position in m-L-SL. By conjugation of additional amino acids to the carboxyl and amino groups of m-L-SL, a complex consisting of six different peptides has been developed. This mixture of peptides, PTC, has shown clinical activity in several human malignancies (Hug et al, 1980; Paccagnella et al, 1986; Zaniboni et al, 1988). In previous investigations with the human melanoma cell line RPMI 8322, PTC as well as some of its individual peptides were more effective than Mel and m-L-SL (Lewensohn et al, 1991*a*; Hansson et al, 1991). Myeloma cells isolated from bone marrow of patients with primary myelomas were more sensitive to PTC than to Mel (Paccagnella et al, 1985). In the human melanoma cell line RPMI 8322, used in the above-mentioned investigation, we found that one of the peptides in PTC, L-prolyl-m-L-sarcolysyl-L-p-fluorophenylalanine (P2), showed a higher toxicity than free m-L-SL as measured by the clonogenic assay (Hansson et al, 1991).

In the present study we show that P2 was the most active component of PTC when tested in a panel of human tumour cell lines. Moreover, P2 was also more active than Mel and m-L-SL against several of the cell lines. Correlation analysis of cell line panel activity patterns demonstrated a close relationship between P2 and Mel, suggesting a similar mode of action. However, unlike Mel and m-L-SL, P2 appeared not to be affected by GSH- and MRP-associated resistance to any greater extent. We have previously found, using a clonogenic assay, that buthionine sulphoximine, which depletes GSH, sensitizes a melanoma cell line to Mel but to a lesser extent to P2 (Hansson et al, 1991). The previous results with BSO as well as the present, showing low RFs for GSH-mediated resistance, may indicate less dependence on

 © Cancer Research Campaign 1998

**Figure 3** Relationship between Mel/P2 $IC_{50}$ ratios and number of doubling times 72 $h^{-1}$ determined by haemocytometer counts in the cell line panel ($n = 10$). $R$ = Pearson's correlation coefficient

**Figure 4** Correlation between log $IC_{50}$ values for Mel and P2 in 49 primary human tumour samples. $R$ = Pearson's correlation coefficient cellular GSH levels for P2 than Mel sensitivity. The explanation for the lack of GSH-mediated resistance in reponse to P2 does not appear to involve intracellular liberation of m-L-SL as this compound shows RFs of similar magnitude to Mel for GSH-associated resistance. Altered substrate recognition of m-L-SL oligopeptides by cellular GSH-dependent enzymes is one potential explanation for the phenomenon. In vitro sensitivity to Mel has previously been noted to result in only a limited increase in toxicity exerted by PTC as compared with Mel in freshly obtained bone marrow myeloma cells from untreated patients.

Interestingly, this finding was contrasted by the relatively pronounced sensitivity to PTC in cell populations with in vitro resistance to Mel (Lewensohn et al, 1991*b*). When comparing Mel, m-L-SL, PTC and P2 on freshly obtained myeloma cells, P2 displayed the highest activity (data not shown).

**Table 5** Correlation of Mel and P2 with standard drugs in haematological tumour cell samples from patients at EDCC

| Compound | Mel (R)[a] | P | P2(R) | P | n |
|---|---|---|---|---|---|
| Doxorubicin | 0.63 | <0.01 | 0.32 | NS | 20 |
| Vincristine | 0.36 | NS[b] | 0.43 | NS | 20 |
| Etoposide | 0.45 | NS | 0.14 | NS | 19 |
| Cytarabine | 0.39 | NS | 0.37 | NS | 20 |
| Cisplatin | 0.58 | <0.05 | 0.24 | NS | 14 |

[a]Pearson's correlation coefficient. [b]NS, not significant ($P > 0.05$).

**Table 6** Comparison of median $IC_{50}$s in CLL and normal PBMC samples for P2 and Mel

| Cell type | $IC_{50}$ P2 | $IC_{50}$ Mel |
|---|---|---|
| PBMC ($n = 5$) | 0.27 | 4.0 |
| CLL ($n = 5$) | 0.07 | 1.4 |
| Ratio PBMC/CLL | 3.9 | 2.8 |

PBMC, peripheral blood mononuclear cell; CLL, chronic lymphocytic leukaemia.

In contrast to proliferating cell lines, human tumour biopsy cells were as a group significantly more sensitive to the cytotoxic activity of P2 than Mel. The reason for this may be related to the low proliferative activity of the primary cultures in the present assay system (Weisenthal et al, 1991) as low-proliferating cell lines also showed higher relative P2 sensitivity. Furthermore, direct manipulation of the proliferative rate of the ACHN cell line produced the corresponding alterations of Mel vs P2 sensitivity. From a clinical point of view, the demonstrated ability of P2 to retain activity against non-cycling cells may be a distinct advantage as the low growth fraction of many solid tumours is a limiting factor for therapeutic responses of most currently used antineoplastic agents. The indications of a wider spectrum of anti-tumour activity and a favourable therapeutic index in vitro as well as the low cross-resistance with standard agents clearly adds to the potential of P2 being a clinically useful anti-tumour agent. What then is the mechanism for increased toxicity of P2 against primary cultures and other non-proliferating cell systems? Although, the drug appears to act mechanistically similar to Mel both in the cell lines and the primary cultures, one may speculate on, at least, two possible explanations. On one hand the effect of a bifunctional alkylating agent is related to the frequency of DNA damage such as DNA cross-links (Lewensohn et al, 1991*a*). The frequency of DNA cross-links may, however, be regulated by DNA repair mechanisms, which at least in some cell lines is correlated with drug sensitivity (Batist et al, 1989). It would then seem possible that a bifunctional alylating agent in the form of an oligopeptide would not be recognized and excised from the DNA by the same repair mechanism as Mel. On the other hand another possible explanation is that of a more effective cellular uptake of the bifunctional alkylator when in the form of an oligopeptide as compared with Mel only. In this context, it is interesting to note that another tripeptide of m-L-SL, 3-(p-fluorophenyl)-L-alanyl-3-[m-bis(2-chloroethyl) aminophenyl]-L-alanyl-L-methionine ethyl ester], PTT.119, has shown increased anti-tumour activity (Yagi et al, 1984, 1988) and the delivery of this peptide into tumour cells was found to be significantly greater than Mel. It was subsequently found that this peptide used multiple transport pathways in L1210 cells (Yagi et al. 1988). Both the above alternatives are currently being explored.

In whole blood P2 is rapidly degraded to m-L-SL, a fact that may limit the activity of the drug in vivo (Ehrsson et al, 1993). This finding indicates that peptidase activity probably degrades the P2 compound intracellularly. Degradation of di-, tri- and tetrapeptides has previously been observed in erythrocytes and leucocytes that have high peptidase activity (Stern et al, 1951). More attempts will be made to characterize exactly the intracellular degradation of P2 and test its efficacy in comparison with m-L-SL in vivo.

In the present study, we used a human cell line panel in combination with a panel of primary tumour cultures from patients for in vitro evaluation of differential drug responses of PTC oligopeptides. In a previous study (Dhar et al, 1996) we showed that the present cell line panel is capable of detecting mechanisms of action of standard drugs in addition to its ability to evaluate sensitivity to drugs to defined types of mechanisms of resistance. Complementary to this, non-clonogenic assays used on fresh primary tumour cultures from patients have been shown to mimic the known clinical activity pattern of standard drugs. We have also previously shown that non-clonogenic cytotoxicity assays such as the FMCA can detect tumour type specific activity retrospectively for a series of standard drugs (Nygren et al, 1994) and prospectively for early phase I–II drugs such as vinorelbine, idarubicin, CdA, gemcitabine, taxol and topotecan (Larsson et al, 1994; Larsson and Nygren, 1994; Csoka et al, 1995; Nygren et al, 1995; Fridborg et al, 1996; Jonsson et al, 1997). Thus, experience gained so far suggests that these model systems may be valid tools for initial predictions of the activity and potential utility of novel anticancer drugs.

In summary, we have demonstrated high anti-tumour activity of a m-L-SL oligopeptide against cell lines and primary cultures of tumour cells from patients. The drug appears to show retained activity against non-proliferating cell systems, shows a positive therapeutic index and demonstrates low levels of cross-resistance with standard drugs. Formal testing of these in vitro predictions will require in vivo testing in relevant tumour models and these studies are currently under way.

### ACKNOWLEDGEMENTS

This work was supported by a grant from the Swedish Cancer Foundation and King Gustav the V Jubilee Fund, Stockholm, Sweden.

### REFERENCES

Batist G, Torres S, Demuys JM, Greene D, Lehnert S, Rochon M and Panasci L (1989) Enhanced DNA cross-link removal: the apparent mechanism of resistance in a clinically relevant melphalan-resistant human breast cancer cell line. *Mol Pharmacol* **36**: 224–230

Bellamy WT, Dalton WS, Gleason MC, Grogan TM and Trent JM (1991) Development and characterisation of a melphalan-resistant human multiple myeloma cell line. *Cancer Res* **51**: 995–1002

Botling J, Liminga G, Larsson R, Nygren P and Nilsson K (1994) Development of vincristine resistance and increased sensitivity to cyclosporin A and verapamil in the human U-937 lymphoma cell line without over expression of the 170 kDa P-glycoprotein. *Int J Cancer* **58**: 269–274

Cole S, Bhardwaj G, Gerlach JH, Almquist KC and Deeley RG (1992) A novel ATP binding cassette transporter gene overexpressed in multidrug resistant human lung tumor cells. *Science* **268**: 1650–1654

Csoka K, Larsson R, Tholander B, Gerdin E, De La Torre M and Nygren P (1994) Cytotoxic drug sensitivity testing of tumor cells from patients with ovarian carcinoma using the fluorometric microculture cytotoxicity assay (FMCA). *Gynecol Oncol* **54**: 163–170

Csoka K, Liliemark J, Larsson R and Nygren P (1995) Evaluation of the cytotoxic activity of Gemcitabine in primary cultures of tumor cells from patients with hematologic or solid tumors. *Semin Oncol* **22**: 47–53

Dalton WS, Durie BG, Alberts DS, Gerlach JH and Cress AE (1986) Characterisation of a new drug-resistant human myeloma cell line that expresses P-glycoprotein. *Cancer Res* **46**: 5125–5130

Danks MK, Yalowich JC and Beck WT (1987) Atypical multidrug resistance in a human leukemic cell line selected for resistance to teniposide (VM-26). *Cancer Res* **47**: 1297–1301

Danks MK, Schmidt CA, Cirtain MC, Suttle DP and Beck WT (1988) Altered catalytic activity of and DNA cleavage by DNA topoisomerase II from human leukemic cells selected for resistance to VM-26. *Biochemistry* **27**: 8861–8869

De Barbieri A (1972) Peptichemio: a synthesis of pharmacological, morphological, biochemical and biomolecular investigations. In: *Proceedings of the Symposium on Peptichemio*, Milan **18**: 13–59

Dhar S, Nygren P, Csoka K, Botling J, Nilsson K and Larsson R (1996) Anti-cancer drug characterisation using a human cell line panel representing defined types of drug resistance. *Br J Cancer* **74**: 888–896

Ehrsson H, Lewensohn R, Wallin I, Hellström M, Merlini G and Johansson B (1993) Pharmacokinetics of peptichemio in myeloma patients: release of M-L-sarcolysin in vivo and in vitro. *Cancer Chemother Pharmacol* **31**: 265–268

Fridborg H, Nygren P, Dhar S, Csoka K, Kristensen J and Larsson R (1996) In vitro evaluation of new anticancer drugs, exemplified by vinorelbine, using the fluorometric microculture cytotoxicity assay on human tumor cell lines and patient biopsy cells. *J Expt Ther Oncol* **1**: 286–295

Gingold N, Pitterman E and Stacher A (1974) Peptichemio in the therapy of malignancies (phase-1-study). The evaluation committee. *Int J Clin Pharmacol Biopharm* **110**: 190–202

Hansson J, Lewensohn R, Ringborg U (1991) Cytotoxicity and DNA cross-linking induced by peptide conjugated m-L-Sarcolysin in human melanoma cells. *Anticancer Res* **11**: 1725–1730

Hug V, Hortobagyi GN, Buzdar AU, Blumenschein GR, Grose W, Burgess MA and Bodey GP (1980) A phase II study of Peptichemio in advanced breast cancer. *Cancer* **45**: 2524–2528

Jonsson E, Fridborg H, Csoka K, Dhar S, Sundström C, Nygren P and Larsson R (1997) Cytotoxic activity of Toptecan in human tumour cell lines and primary cultures of human tumour cells from patients. *Br J Cancer* **76**: 211–219

Larsson R and Nygren P (1994) Cytotoxic activity of topoisomerase II inhibitors in primary cultures of human tumour cells from patients with human hematologic and solid tumors. *Cancer* **74**: 2857–2862

Larsson R, Kristensen J, Sandberg C and Nygren P (1992) Laboratory determination of chemotherapeutic drug resistance in tumor cells from patients with leukemia using a fluorometric microculture cytotoxicity assay (FMCA). *Int J Cancer* **50**: 177–185

Larsson R, Fridborg H, Liliemark J, Csoka K, Kristensen J, De La Torre M and Nygren P (1994) In vitro activity of 2-chlorodeoxyadenosine (CdA) in primary cultures of human hematological and solid tumors. *Eur J Cancer* **30A**: 1022–1026

Lewensohn R, Ehrsson H, Hansson J and Ringborg U (1991*a*) Increased toxicity and DNA cross-linking by peptide bound m-L-Sarcolysin (Peptichemio) as compared to melphalan and m-L-Sarcolysin in human melanoma cell lines. *Anticancer Res* **11**: 321–324

Lewensohn R, Fernberg JO, Ehrsson H and Merlini G (1991*b*) Efficacy of peptide bound m-L-sarcolysin (Peptichemio) on melphalan resistant human myeloma cells in vitro. *Med Oncol Tumor Pharmacother* **8**: 265–269

Merlini G, Gobbi GP, Riccardi A, Riva G, Sardi C and Perugini S (1982) Peptichemio induction therapy in myelomatosis. *Cancer Chemother Pharmacol* **8**: 9–16

Mirski SE, Gerlach JH and Cole SP (1987) Multidrug resistance in a human small cell cancer cell line selected in adriamycin. *Cancer Res* **47**: 2594–2598

Mulcahy RT, Bailey HH and Gipp JJ (1994) Up-regulation of gamma-glutamylcysteine synthetase activity in melphalan-resistant human multiple myeloma cells expressing increased glutathione levels. *Cancer Chemother Pharmacol* **34**: 67–71

Nygren P and Larsson R (1990) Verapamil and cyclosporin A sensitize human kidney tumor cells to vincristine in absence of membrane P-glycoprotein and without apparent changes in the cytoplasmic free $Ca^{2+}$ concentration. *Biosci Rep* **10**: 231–237

© Cancer Research Campaign 1998

Nygren P. Kristensen J. Sundström C. Lönnerholm G. Kreuger A and Larsson R (1992) Feasibility of the fluorometric microculture cytotoxicity assay (FMCA) for cytotoxic drug sensitivity testing of tumor cells from patients with acute lymphoblastic leukemia. *Leukemia* **6**: 1121–1128

Nygren P. Fridborg H. Csoka K. Sundström C. De La Torre M. Kristensen J. Bergh J. Hagberg H. Glimelius B. Rastad J. Tholander B and Larsson R (1994) Detection of tumor-specific cytotoxic drug activity in vitro using the fluorometric microculture cytotoxicity assay and primary cultures of tumor cells from patients. *Int J Cancer* **56**: 715–720

Nygren P. Csoka K. Jonsson B. Fridborg H. Bergh J. Hagberg H. Glimelius B. Brodin O. Tholander B. Kreuger A. Lönnerholm G. Jakobsson Å. Olsen L. Kristensen J and Larsson R (1995) The cytotoxic activity of Taxol in primary cultures of tumor cells from patients is partly mediated by Cremophore EL. *Br J Cancer* **71**: 478–481

Paccagnella A. Tredese F. Salvagno L. Brandes A. Sileni VC. Daniele O. Fornasiero A. Fosser V. Nicoletto O. Maggino T and Florentino MV (1985) Peptichemio in pretreated patients with ovarian cancer. *Cancer Treat Rep* **69**: 17–20

Paccagnella A. Salvagno L. Chiarion-Sileni Il V. Boiznella S. De Besi P. Frizzarin M. Pappagallo GL. Fosser VP. Fornasiero A. Segati R and Florentino MV (1986) Peptichemio in pretreated patients with plasma cell neoplasms. *Eur J Cancer Clin Oncol* **22**: 1053–1058

Perugini S. Bobbio PE. Ghizzi A. Riccardi A. Martinotti A and Merlini P (1976) Peptichemio in the treatment of plasma cell leukemia. *Haematologica* **61**: 248–249

Stern K. Birmingham MK. Cullen A and Richer R (1951) Peptidase activity in leucocytes, erythrocytes and plasma of young adult and senile subjects. *J Clin Invest* **30**: 84–89

Weisenthal LM. Dill P and Birkhofer M (1991) Accurate identification of disease-specific activity of antineoplastic agents with an in vitro fresh tumor assay measuring killing of largely non-dividing cells. *Proc Am Assoc Cancer Res* **32**: 384

Yagi MJ. Bekesi JG. Daniel MD. Holland JF and De Barbieri A (1984) Increased cancericidal activity of PTT.119, a new synthetic bis-(2-chloroethyl)amino-L-phenylalanine derivative with carrier amino acids. *Cancer Chemother Pharmacol* **12**: 70–76

Yagi MJ. Scanlon KJ. Chin SE. Holland JF and Bekesi JG (1988) Multiple transport pathways for L1210 cells: uptake of PTT.119, a bifunctional alkylator with carrier amino acids. *Chemotherapy* **34**: 235–247

Zaniboni A. Simoncini E. Marpicati P. Montini E. Rossi G and Marini G (1988) Peptichemio, teniposide and high-dose dexamethasone: a new active combination for relapsing and refractory multiple myeloma. A pilot study. *Anticancer Res* **8**: 125–128

© Cancer Research Campaign 1998